# Editorial: The psychological process of stereotyping: Content, forming, internalizing, mechanisms, effects, and interventions

**DOI:** 10.3389/fpsyg.2022.1117901

**Published:** 2023-01-05

**Authors:** Baoshan Zhang, Yibo Hu, Fengqing Zhao, Fangfang Wen, Junhua Dang, Magdalena Zawisza

**Affiliations:** ^1^School of Psychology, Shaanxi Normal University, Xi'an, China; ^2^School of Education, Zhengzhou University, Zhengzhou, China; ^3^School of Psychology, Central China Normal University, Wuhan, China; ^4^Department of Surgical Sciences, Uppsala University, Uppsala, Sweden; ^5^Department of Psychology, Anglia Ruskin University, Cambridge, United Kingdom

**Keywords:** stereotyping, forming of social stereotypes, consequences of negative stereotypes, neurocognitive mechanisms, interventions

Stereotype is a pervasive and persistent human tendency that stems from a basic cognitive need to categorize, simplify, and process the complex world. This tendency is a precondition for social bias, prejudice, and discrimination. Amid the COVID-19 outbreak, the discrimination, exclusion, and even hostility caused by stereotypes have increasingly become an important social issue that concerns political and social stability. Therefore, the current issue focuses on a broad spectrum of research addressing four main themes: (1) the psychological processes involved in forming and internalizing social stereotypes, (2) the negative consequences of stereotypes, (3) the neurocognitive mechanisms underlying stereotypes, and (4) the interventions addressing the consequences of negative stereotypes in this era with changes and challenges. Specifically, the Research Topic consists of 13 papers by 54 scholars that target stereotypes among different social groups, including males and females, older people and young generation, minority races, people living with HIV/AIDS (PLWHA), people with mental health problems, juvenile transgressors, refugees, and Asian-Americans during COVID-19 outbreak. These studies are conducted in culturally diverse countries including Brazil, China, Germany, Hungary, and the USA, contributing to a more holistic picture of contemporary stereotypes.

## 1. The forming of social stereotypes

Negative stereotypes from the public may be influenced by our knowledge about and psychological distance to the target group, beliefs of group malleability, beliefs in the implicit change of traits, and moral values. For instance, Caldas et al. tested whether people's knowledge and proximity to the circumstances associated with juvenile transgression would influence their opinions about the proposal for reducing the age of criminal majority in Brazil. They investigated the passers-by in a public square and workers from the juvenile justice courts and found that people were more likely to hold negative stereotypes of juvenile delinquents if they were far from them. Paskuj and Orosz focused on the refugees as the most typically vulnerable group in turbulent international times, and they found that group malleability beliefs were negatively linked to dehumanization tendencies and threats perceived from migrants in Hungary. Protzko and Schooler examined a more general negative stereotype of youth also known as the “kids these days effect” (KTD effect). In two studies with American adults, belief in whether a trait changes over the lifespan was associated with such prejudices. In addition, Lai et al. focused on three cues linked to women's perceived high long-term mating value and reported that Chinese women displaying “sexually attractive” cues were perceived to have lower moral values. Moreover, they were stereotyped as having lower levels of humanness than women displaying “beautiful” facial cues or “virtuous” behavioral cues, which in turn led to lower mating opportunity.

Culture also plays an essential role in stereotype formation. Li M. et al. targeted stereotypes toward high-power individuals and revealed that people influenced by Confucianism held positive stereotypes of competence and warmth for senior high-power individuals. This finding is inconsistent with the traditional proposition that high-power individuals tend to be stereotyped as having high competence and low warmth. This might be because high-power individuals under Confucian culture are expected to have great social responsibility and concern for the wellbeing of others. Furthermore, new stereotypes emerged as a result of COVID-19 in the global context. COVID-19 is a threat to physical health, and mental health, and various reports have indicated that COVID-19 is closely related to stigma and discrimination. Two studies examined the stereotypes related to COVID-19. Zhao et al. found that the prevalence of COVID-19-related negative stereotypes was low in China. Besides, the more people know about COVID-19, the fewer negative stereotypes associated with COVID-19 they reported. Daley et al. on the other hand reported that Asian-Americans were facing increasing challenges from different ethnic groups on social issues related to COVID-19 in the United States, and the increasing tendency to blame China for the pandemic was associated with stereotyping Asian people as more foreign.

## 2. The consequences of negative stereotypes

People's negative stereotypes will influence their behavioral inclinations toward the target groups, and even the law-making at a general level. For instance, Wen et al. tested space-related stereotypes associated with people living with HIV/AIDS (PLWHA). They found that people who held negative stereotypes toward the spaces occupied by PLWHA were more resistant to visit such spaces, and people's threat perception and community evaluation mediated the effects of such space-related stereotypes on community-approaching willingness. In addition, Caldas et al. found that the more distant people were from juvenile transgressors, the more they held negative stereotypes toward juvenile transgressors and agreed with the law-making proposal for reducing the age of criminal conviction in Brazil.

Vulnerable groups may internalize the negative stereotypes and be influenced by them. Gärtner et al. tested the self-stereotyping of people with mental illness and found that negative stereotypes of their warmth and competence dimensions led them to develop negative emotions and thus exhibit higher levels of active or passive self-harm than mentally healthy people. In addition, Li J. et al. were interested in the gender self-stereotyping among college students and noted that gender self-stereotyping was positively correlated with relational and personal self-esteem and further correlated with higher life satisfaction only in the male sample. That is, gender self-stereotyping was associated with a higher level of self-esteem and life satisfaction among male students, while this effect did not hold for women.

## 3. The neurocognitive mechanisms of stereotypes

The neurocognitive mechanisms of stereotypes were explored by Wu and Zhao. They used RS-fMRI degree centrality (RSDC), a graph theory-based network analysis, to detect how negative stereotypes work in the brain. In a test of math-related stereotypes among female university students, they found that the RSDC of different brain regions was affected, reflecting that stereotypes are the result of the action of the brain network as a whole. For instance, a decrease in RSDC in the left hippocampus is a response to stereotype-related stress, and an increase in RSDC in the posterior parietal region (PPC) is a reflection of self-relevant processes induced by stereotypes.

## 4. The interventions addressing the consequences of negative stereotypes

Finally, two studies tested interventions against negative stereotypes *via* intergenerational contact and cognitive training. Long et al. found that simply intergenerational contact, or even just imagining it, reduced negative stereotypes of older people and increased perspective-taking toward older people among young adults. Chen et al. used the traditional IAT to compare the effect of multiple vs. single cognitive training on aging stereotypes in 12–13-year-olds. They found that multiple training tasks and additional intervention training sessions are recommended as they could significantly prolong the positive effects of the intervention.

Overall, these 13 papers discussed various aspects of stereotype formation, consequences, mechanisms, and interventions. We hope these papers will inspire future researchers in developing theories and conducting new interventions against negative effects of stereotypes. Since the current era of “black swan incidents” and related social challenges create perfect conditions for stereotypes to thrive and intensify, researchers should continue exploring the psychological mechanisms behind emerging social stigma and negative stereotypes. Especially, the development of neuroscience will provide further opportunities to study the brain mechanisms of stereotypes from a more microscopic perspective. This combined with macroscopic psychosocial mechanisms will provide new ways of addressing the severe dangers of negative stereotypes across contexts, countries and times and benefit targeted interventions and policy making.

## Author contributions

All authors listed have made a substantial, direct, and intellectual contribution to the work and approved it for publication.

